# Supplementary material for: Trigger factor chaperone acts as a mechanical foldase
Source: Nat Commun. 2017 Sep 22;8:668. doi: 10.1038/s41467-017-00771-6 (PMC5610233; doi:10.1038/s41467-017-00771-6)
Supplement: Supplementary file 1 — Supplementary Information [file 41467_2017_771_MOESM1_ESM.pdf]

Description of Supplementary Files

File name: Supplementary Information
Description: Supplementary figures, supplementary tables, supplementary discussion and supplementary references.

File name: Peer review file

Supplementary Discussion

Unfolding of protein L in presence and absence of TF

To investigate the effect of TF on the mechanical unfolding of octamer Protein L, Protein L molecules are unfolded in two different protocols, by applying a constant force (force-clamp) and applying a linearly increasing force (force-ramp). Supplementary Figure 1A shows three typical force-clamp unfolding trajectories at different forces (15, 26 and 35 pN). Upon force application, the unfolding of each domain appears as a length step whose elongation depends on the force, following the Freely-Jointed Chain polymer model[1, 2]. We characterize the unfolding kinetics by Mean First Passage Time (MFPT) measurements to the fully unfolded octamer. Supplementary Figure 1B shows the unfolding rates (inverse of MFPTs) with (black) and without (red) TF, plotted in a semi logarithmic scale. Clearly, the presence of the TF does not affect the unfolding rate, suggesting that the native state is unaffected by it.

In order to further confirm this observation, we perform force ramp experiments, where the force is linearly increased, stretching the polyprotein and unfolding the domains with an increasing probability. Supplementary Figure 1C shows a typical extension versus time trajectory. The extension of the polyprotein increases with time, and each unfolding event appears as a discrete length step, which occurs at different forces. Supplementary Figure 1D shows the unfolding force histograms in presence (red) and absence (black) of TF. Each histogram has been built from a total of 200 unfolding events. Clearly, the unfolding of Protein L is unaffected by the presence of TF, as reflected in the overlapping of both histograms.

The chaperone activity of TF in the near-zero force regime

In order to investigate the activity of TF in the near-zero force regime, we design the protocol depicted in Supplementary Figure 2, Panel A. After the fingerprint pulse, where all eight domains unfold (N_f=8 number of domains), we allow the molecule to fold for different quench times t_{quench} at 0.7 pN, in absence and presence of TF, subsequently unfolding the polyprotein in the probe pulse. There, the successfully folded domains would unfold as measured steps, so that

we recover N_p. Next, we allow the molecule to fold at 4 pN for long time ~100 s, in order to repeat the pulse protocol (not shown in figure). This protocol allows us to calculate the fraction of folded domains as a function of the quench time $N_p/N_f\,(t_{quench})$, in presence and absence of TF. Supplementary Figure 2, Panel B shows this fraction for quench times of t_{quench}=1,2,3,5,10,15,20,30 seconds. Note that, before lowering the force from 45 pN, to 0.7 pN, we do a pre-quench at 13 pN for 3 seconds, where the folding probability is zero. This allows us to minimize the error in the time upon force changes, due to the movement of the magnets, which is larger for larger changes (here from 45 pN to 0.7 pN the error can be up to 0.3 s, whereas between 13 and 0.7 pN, the error is less than 0.1 s).

After long quench times (20-30 seconds), protein L domains fold successfully $N_p/N_f\,(t_{quench})$~1 in the absence of TF, while TF shows a significantly lower fraction ~0.65, meaning that at very low forces, TF hinders refolding, working thus as a holdase, as previously reported.

Folding probability change in presence of BSA

We perform a control experiment monitoring the folding probability at 7.4 pN at increasing concentration of bovine serum albumin protein (BSA). Supplementary Figure 5 compares the change in the folding probability of protein L at 7.4 pN in presence of TF and BSA. While TF leads to an evident increase of the folding probability, ranging from 0.43 to 0.90, when the TF concentration is 1 uM, BSA does not modulate the folding probability, which remains at its basal value of 0.43 regardless of the BSA presence. This result directly indicate that it is the TF activity which affects the folding properties of Protein L under force, and not any crowding effect due to the presence of the concentration of an additional protein.

Sigmoid fits to the folding probability

The folding probability as a function of the force is fitted with a sigmoid equation:

$$FP(F) = B + \frac{M}{1+e^{-(F-F_m)/r}} \qquad (1)$$

where B and M are respectively the base and maximum value of the sigmoid (here fixed to B=1 and M=-1), F_m is the force at which the half change is achieved and r is the rate. Obtained values for the fits shown in Supplementary Table 1.

Supplementary Table 1: Folding probability fitting parameters in presence and absence of Trigger Factor.

	B	M	F_m (pN)	r (pN)
**TF**	1 (fixed)	-1 (fixed)	9.16±0.06	0.55±0.05
**NoTF**	1 (fixed)	-1 (fixed)	7.98±0.09	0.93±0.09

Exponential fits to MFPT

The *MFPT* as a function of the force is fit with an exponential growth function:

$$MFPT(F) = Ae^{-F/r} \quad (2)$$

Supplementary Table 2. *MFPT* fitting parameters in presence and absence of Trigger Factor.

	A (s)	r (pN)
**TF**	0.117±0.055	1.112±0.123
**NoTF**	0.001±0.000	0.493±0.093

Hill fits to the concentration-dependency study

The change of folding probability with the TF concentration is fit with a Hill equation:

$$P_f([TF]) = B + \frac{M-B}{1+\left({[TF]_m}/{[TF]}\right)^r} \quad , \quad (3)$$

where B and M are respectively the base and maximum P_f, $[TF]_m$ is the concentration of the half change and r the rate. The obtained values from the fits are:

Supplementary Table 3: Fitting parameters of folding probability at different concentration

Force (pN)	B	M	r	$[TF]_m$ (uM)
**6.2**	0.87±0.02	0.96±0.03	4.00±0.12	0.59±0.08
**7.4**	0.64±0.01	0.96±0.05	5.35±0.22	0.89±0.17
**8.1**	0.43±0.01	0.91±0.01	7.90±0.25	0.90±0.26
**8.4**	0.45±0.06	0.85±0.06	1.98±0.67	2.67±0.59
**8.9**	0.22±0.03	0.70±0.08	1.00±0.31	27.54±2.40
**11.7**	0.00	0.00	0.00	0.00

Folding probability supplementary tables

We include the tables with the populations of each state at each force and the calculated folding probability using Eq. (1) based on method section.

Supplementary Table 4: State occupation at F=5.2 pN with and without TF.

F=5.2 pN			
No TF		TF	
State i	π_i	State i	π_i
8	0.147	8	1.000
7	0.809	7	0.000
6	0.04	6	0.000
5	0.001	5	0.000
4	0.000	4	0.000
3	0.000	3	0.000
2	0.000	2	0.000
1	0.000	1	0.000
0	0.000	0	0.000
P_f =0.889		P_f =1.000	

Supplementary Table 5: State occupation at F=6.2 pN with and without TF.

F=6.2 pN			
No TF		TF	
State i	π_i	State i	π_i
8	0.233	8	0.855
7	0.577	7	0.137
6	0.141	6	0.008
5	0.034	5	0.000
4	0.006	4	0.000
3	0.000	3	0.000
2	0.000	2	0.000
1	0.000	1	0.000
0	0.000	0	0.000
P_f=0.877		P_f =0.981	

Supplementary Table 6: State occupation at F=7.4 pN with and without TF.

F=7.4 pN			
No TF		TF	
State i	π_i	State i	π_i
8	0.030	8	0.501
7	0.185	7	0.377
6	0.294	6	0.075
5	0.238	5	0.044
4	0.212	4	0.003
3	0.042	3	0.000
2	0.015	2	0.000
1	0.013	1	0.000
0	0.001	0	0.000
P_f =0.688		P_f=0.916	

Supplementary Table 7: State occupation at F=8.1 pN with and without TF.

F=8.1 pN			
No TF		TF	
State i	π_i	State i	π_i
8	0.004	8	0.245
7	0.050	7	0.348
6	0.106	6	0.234
5	0.226	5	0.121
4	0.296	4	0.050
3	0.209	3	0.003
2	0.108	2	0.000

1	0.001	1	0.000
0	0.000	0	0.000
P_f =0.522		P_f =0.826	

Supplementary Table 8: State occupation at F=8.5 pN with and without TF

F=8.5 pN			
No TF		TF	
State i	π_i	State i	π_i
8	0.009	8	0.243
7	0.05	7	0.427
6	0.104	6	0.178
5	0.164	5	0.094
4	0.201	4	0.058
3	0.215	3	0.000
2	0.153	2	0.000
1	0.088	1	0.000
0	0.023	0	0.000
P_f =0.459		P_f =0.830	

Supplementary Table 9: State occupation at F=8.9 pN with and without TF.

F=8.9 pN			
No TF		TF	
State i	π_i	State i	π_i
8	0.000	8	0.094
7	0.000	7	0.131
6	0.008	6	0.229
5	0.035	5	0.182
4	0.100	4	0.278
3	0.180	3	0.086
2	0.317	2	0.000
1	0.231	1	0.000
0	0.128	0	0.000

P_f =0.254	P_f =0.666

Supplementary Table 10: State occupation at F=9.7 pN with and without TF.

F=9.7 pN			
No TF		TF	
**State i**	**π_i**	**State i**	π_i
8	0.000	8	0.000
7	0.000	7	0.000
6	0.000	6	0.000
5	0.003	5	0.000
4	0.013	4	0.057
3	0.054	3	0.156
2	0.112	2	0.146
1	0.219	1	0.334
0	0.600	0	0.208
P_f =0.084		P_f =0.190	

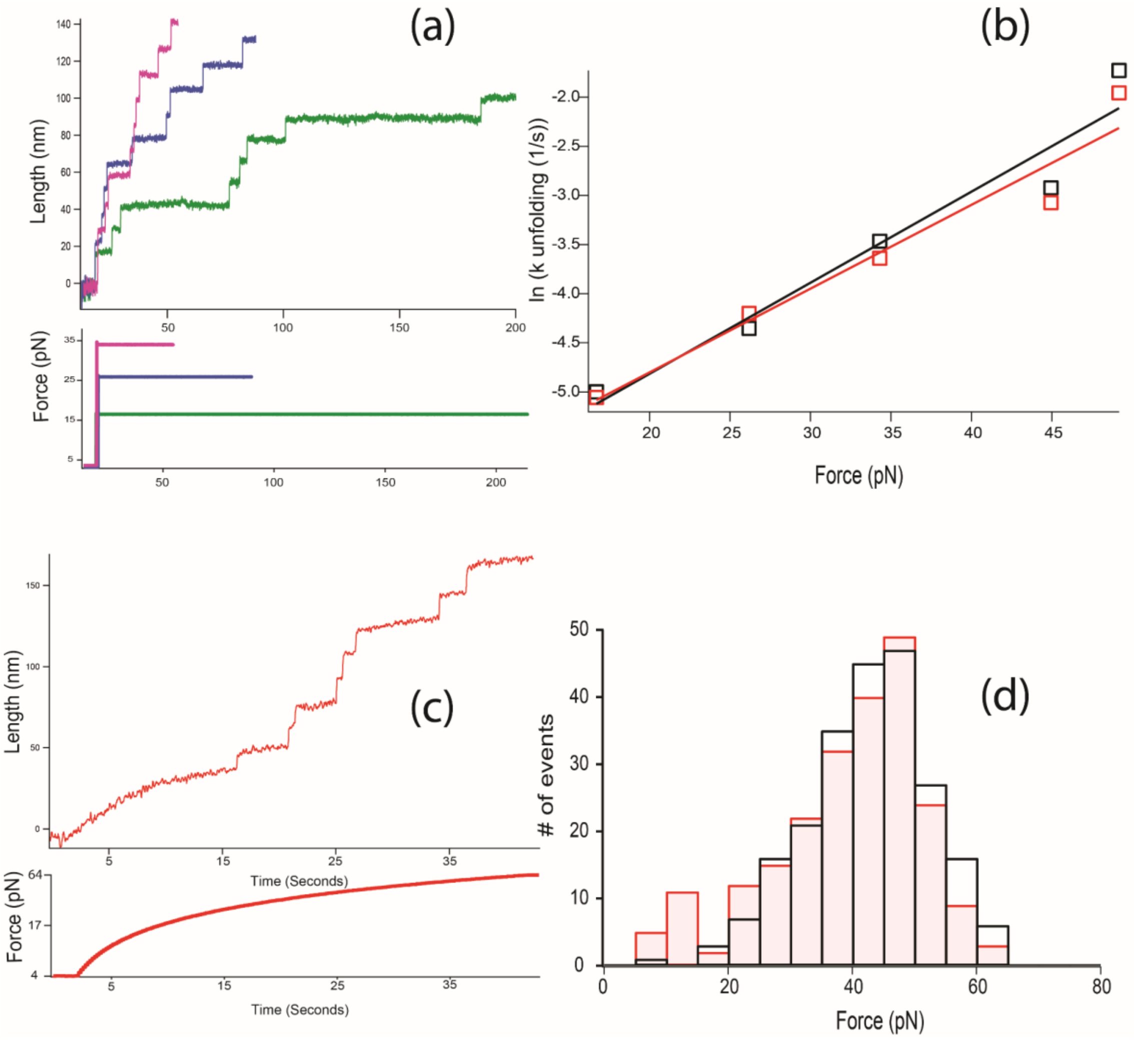

Supplementary Figure 1: Compared unfolding dynamics in presence (red) and absence (black) of Trigger factor. **(A)** Unfolding traces of eight-domain Protein L measured at three different constant magnetic forces, 16 pN (green), 26 pN (blue) and 35 pN (pink). **(B)** Unfolding rates as obtained from Mean First Passage Time (MFPT) measurements to the fully unfolded octamer of protein L in presence (red) and absence (black) of 500 uM Trigger Factor, showing no effect on the unfolding dynamics of protein L. Data points are averages over more than ten experiments. **(C)** Mechanical unfolding of protein L using a force-ramp protocol, where the force increases at a constant rate of 1.5 pN/s. Each step increase in length marks the force at which the

unfolding event occurred. **(D)** Unfolding force histogram of protein L in presence (red) and absence (black) of TF. Both histograms overlap, confirming that Trigger Factor does not affect the unfolding kinetics of protein L.

A

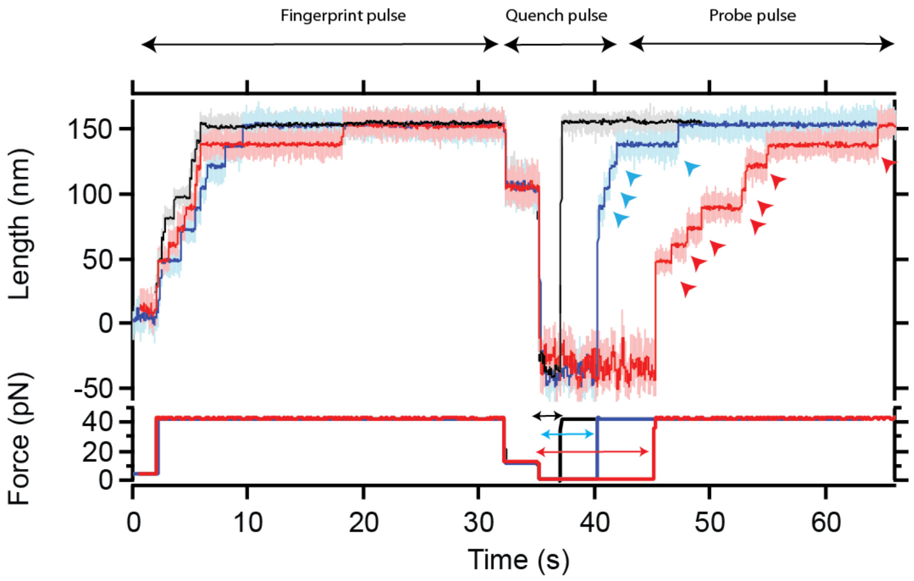

B

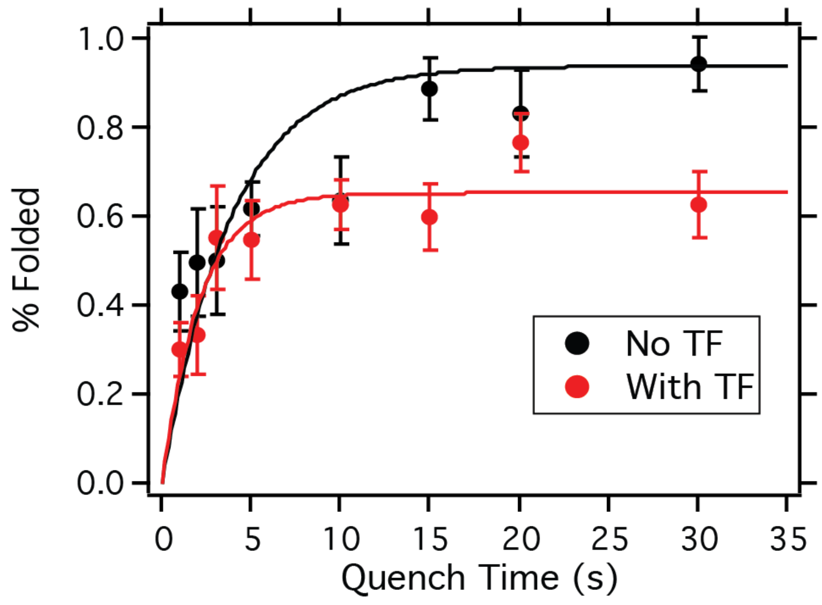

Supplementary Figure 2: Activity of TF after quench pulses to near-zero forces: (A) Protocol used to investigate the effect of TF on the folding properties of protein L in the near-

zero regime. **(B)** Fraction of folded domains as a function of the quench time at 0.7 pN. TF hinders the refolding transition as it lowers the fraction of folded domains at long quench times. Data points are averages over more than ten experiments and five molecules. Errors bars are s.e.m.

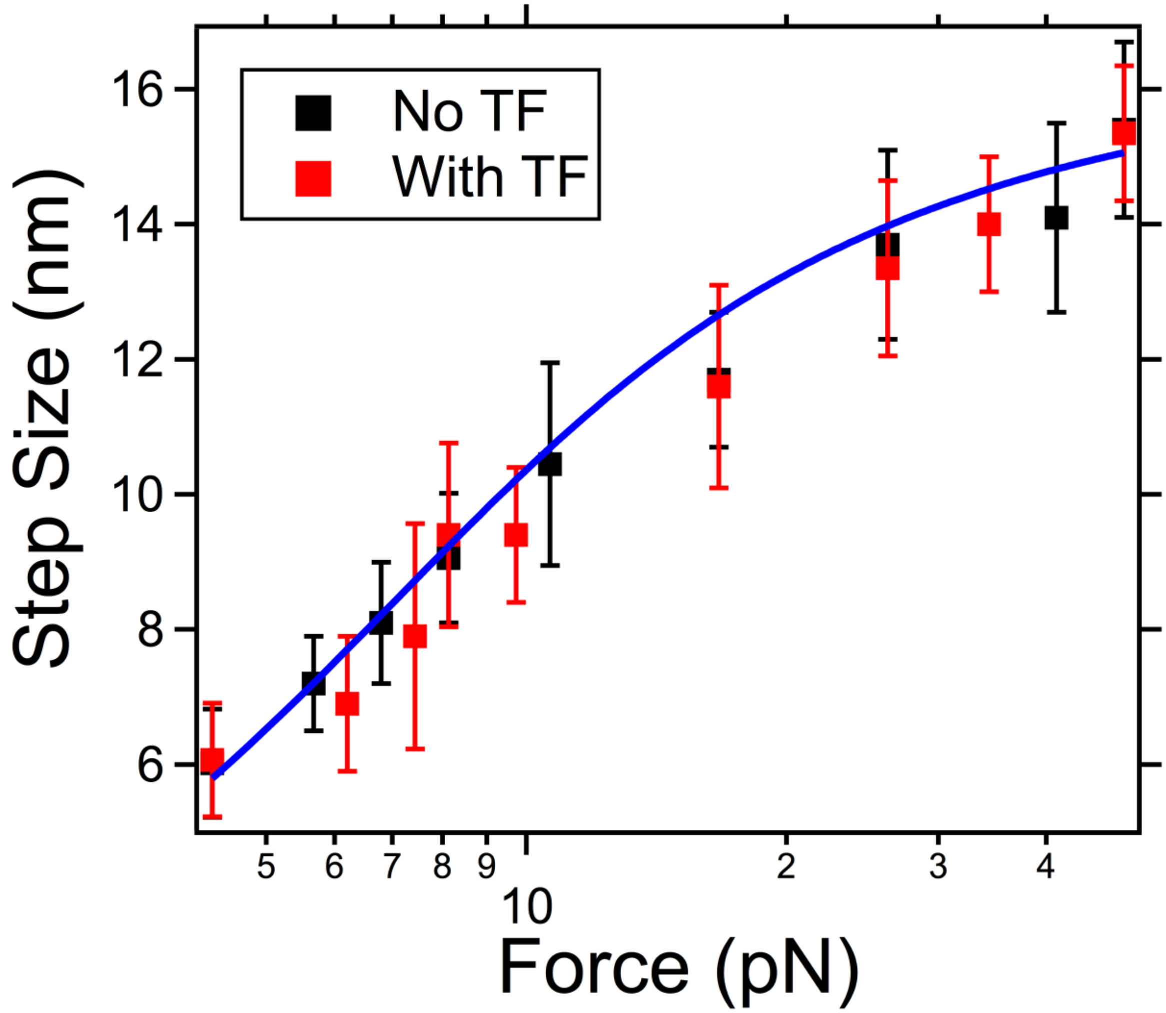

Supplementary Figure 3: Step-size as a function of the force in presence (red) and absence (black) of TF. TF does not induce any appreciable change on the polymer properties of protein L domains, and both sets of data can be represented by a Freely-Jointed chain model with contour length L_c=16.3 nm and Kuhn length K=1.1 nm. Data points are averages over more than 100 step sizes, and five molecules. Error bars are s.e.m.

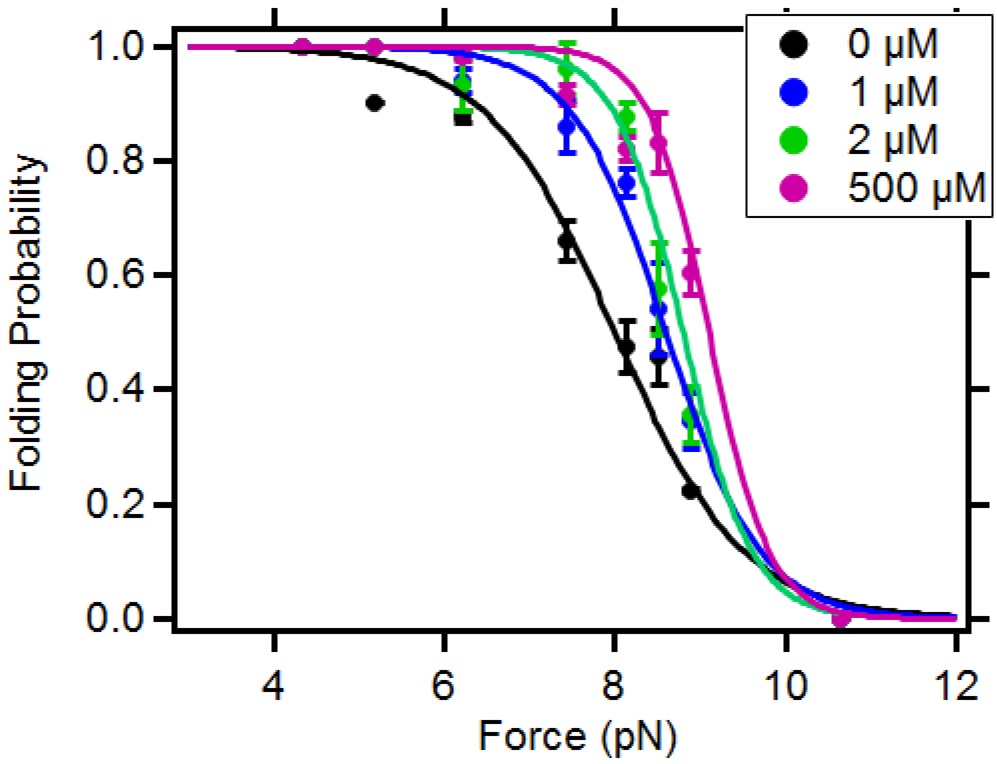

Supplementary Figure 4: Folding probability as a function of the TF concentration. The folding probability as a function of the pulling force is plot for four different TF concentrations (0, 1, 2 and 500 μM). This plot highlights that the biggest influence of TF to the folding probability occurs up to 1 uM, where the effect saturates at most forces, except for the higher ones (over 8.5 pN), which need a greater increase in the concentration to show the maximal folding probability increase. Data points are calculated as described above, using >3000 seconds, and over more than three molecules per force. Errors bars are s.e.m.

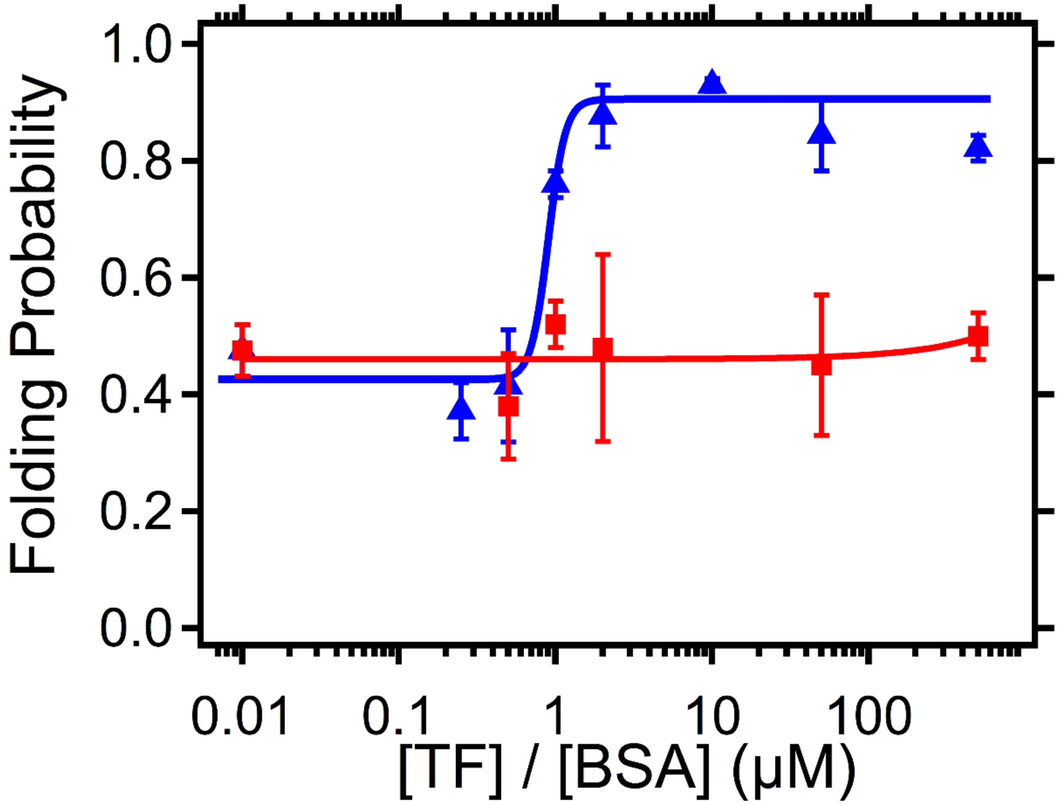

Supplementary Figure 5: Folding probability of Protein L at 7.4 pN as a function of Trigger Factor (blue) and BSA (red) concentration. Trigger Factor increases the folding probability of protein L while BSA has no apparent effect. Data points are calculated as described above, using >3000 seconds, and over more than three molecules per force. Errors bars are s.e.m.

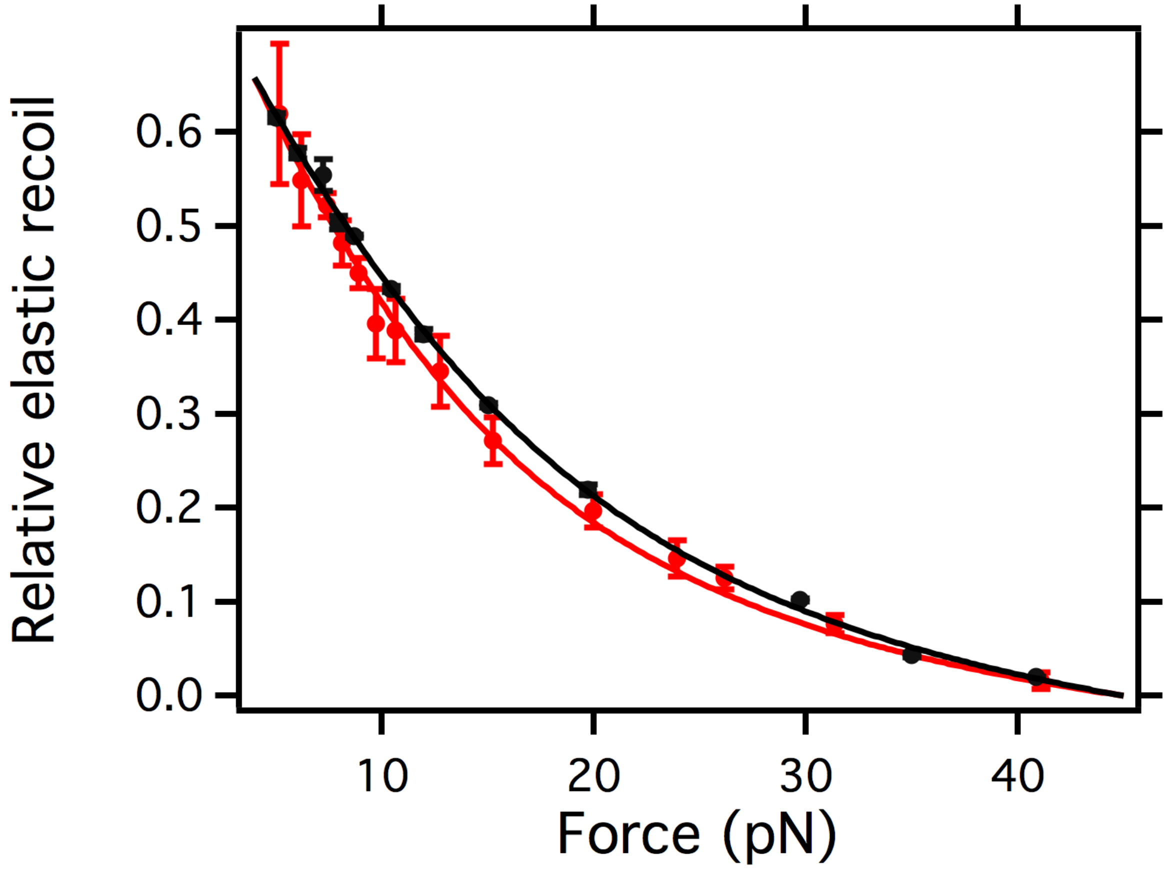

Supplementary Figure 6: Relative elastic recoil in presence (red) and absence (black) of TF. The unfolded molecule can be modeled with a Freely Jointed Chain with Kuhn length of K=0.6 +-0.5 nm and K=0.5+-0.4 nm, respectively. TF does not induce any appreciable change in the polymer properties of the unfolded molecule. Data points are averages over more than five molecules. Error bars are s.e.m.

Supplementary References

1. Valle-Orero J, Eckels EC, Stirnemann G, Popa I, Berkovich R, Fernandez JM. The elastic free energy of a tandem modular protein under force. *Biochemical and biophysical research communications* **460**, 434-438 (2015).

2. Popa I, *et al.* A HaloTag Anchored Ruler for Week-Long Studies of Protein Dynamics. *Journal of the American Chemical Society* **138**, 10546-10553 (2016).
